# Mobile Phone Short Message Service (SMS) for Weight Management in Iranian Overweight and Obese Women: A Pilot Study

**DOI:** 10.1155/2013/785654

**Published:** 2013-09-17

**Authors:** Somayeh Faghanipour, Eftekharalsadat Hajikazemi, Soghra Nikpour, Shabnam al-Sadat Shariatpanahi, Agha Fatemeh Hosseini

**Affiliations:** ^1^School of Nursing and Midwifery and Center for Nursing Care Research, Tehran University of Medical Sciences, P.O. Box 1419733171, Tehran, Iran; ^2^Center for Nursing Care Research, Tehran University of Medical Sciences, P.O. Box 1995614111, Tehran, Iran; ^3^School of Nursing and Midwifery, Tehran University of Medical Sciences, P.O. Box 1419733171, Tehran, Iran; ^4^School of Health Management & Medical Information Science, Tehran University of Medical Sciences, P.O. Box 1995614111, Tehran, Iran

## Abstract

We conducted a text message-based intervention for weight management over three months by two months intervention and one month wash-out period. In a quasi-experimental study with control (*n* = 40) and experimental group (*n* = 40), 80 overweight and obese employed women were entered. Participants were recruited via announcement. All subjects attended a face-to-face information session and received a booklet that contained food calorie chart and strategies and recommendations for weight management. The experimental group received text messages (SMS) about weight management twice a day for two months, in addition to the information and the booklet which they had received in the information session. Also, the experimental group was instructed to weekly self-weight and to send the data to the principle researcher. All subjects were measured for baseline and secondary weight in a standardized manner by a nurse, and the data were compared between the two groups. Experimental group lost more weight than the control group (1.5 kg difference, *P* = 0.018). Text messaging seems to be an effective channel of communication for weight management in Iranian overweight and obese women. The clinical trial registration number is IRCT201204029360N1.

## 1. Introduction

In recent decades, the prevalence of obesity has increased to an alarming degree throughout the world. According to World Health Organization's (WHO) report, developing countries parallel to developed world are experiencing a growing trend in the incidence of obesity and overweight. They are joining the global pandemic of obesity because of modernization and urbanization [1, 2]. Overweight and obesity are important risk factors for diabetes type II, insulin resistance, cardiovascular disease, hypertension, selected cancers, and premature death [3]. Obesity is closely associated with some psychological disturbances such as poor body image and self-esteem, depression, and anxiety, particularly in women [4, 5]. From 1999 to 2008, the prevalence of overweight Iranian women adults has dramatically increased from 40.6% to 61%. In the same time period, the prevalence of obese Iranian women has risen from 14.17% to 29.5%. [6]. This increase has been more common among Iranian women than Iranian men [7, 8].

Despite the numerous clinical and commercial weight loss programs in Iran, many individuals hardly use such programs because of various limitations including time, cost, transportation, beside, women who work outside the home have more constraints than others. Therefore, evaluation of different approaches is necessary to find an effective, acceptable, economic, and continuous program for modifying behavior and lifestyle factors associated with obesity [9]. Cell phone's short message service (SMS) is a communicative tool which potentially modifies health behavior for several reasons [10]; the cost is relatively low, its use is widespread, and it is applicable to every model of mobile phone [11]. 

Although the application of text messaging for behavior change is in its infancy, there is some supportive evidence for changing behaviors and lifestyle, managing diseases, or preventing from them [9, 12–17]. In a randomized controlled trial in the USA, a mobile phone-based intervention helped overweight and obese individuals lose weight over 4 months. In a controlled study among adults in the UK, a combination of a fully automated web-based planning tool with mobile text reminders led to a lower self-reported consumption of high-fat foods than in a control condition [18]. Text messaging can be used in developing countries where people may not have access to expensive technologies. Because of low Internet penetration rate, text messaging can be considered as an effective tool for addressing health behaviors in Iran. To date and our knowledge, only one study has investigated the effect of SMS on health in Iran [12], and there have been no studies on the use of text messaging to address overweight and obesity among Iranian adults. The aim of the present study was to assess the effectiveness of text messaging as an intervention to help Iranian overweight and obese women manage their weight.

## 2. Materials and Methods

### 2.1. Participants

The study included 80 overweight and obese employed women. In this quasi-experimental study with control and experimental groups, the subjects were selected from administrative departments of Tehran University of Medical Sciences, Tehran, the capital of Iran. As inclusion criteria, they had their own cell phone which could send and receive SMS in Persian language, they were 25 to 55 years old and were overweight or obese (BMI ≥ 25), they did not take medications known to cause weight gain, they were not pregnant or breastfed or in 6-months postpartum period, they were not under specific diet or exercise program. The subjects were excluded if they had a serious physical or mental illness and got pregnant, or entered in a special diet or exercise program during the study. Participants were recruited via announcement posted on bulletin board. Interested volunteers were screened for meeting inclusion criteria, and finally, eligible volunteers were selected. For avoiding information contamination, participants were selected from two separated administrative departments. Those departments were randomly assigned to either the control or experimental group. The subjects in both experimental (*n* = 40) and control groups (*n* = 40) were not informed of their group allocation status and were blinded to the group's assignment. 

Medical Research Ethics Committee of Tehran University of Medical Sciences approved the research protocol. All subjects signed informed consent form prior participation in the study. Confidentiality was guaranteed to the participants. The participants were informed that they could withdraw from the study at any time. They did not receive any reward for participating in the study. 

### 2.2. Intervention

This pilot study lasted for three months (two months intervention and one month wash-out period). Total body weights and heights were obtained with a digital adult calibrated scale (model 708, Seca, Germany, 0.01 kg) and a wall-mounted stadiometer (model 206 Seca, 0.1 cm), respectively, by a community health nurse. They were wearing light clothing and no shoes when they were being measured. Body mass index (BMI) was calculated. All subjects in both groups attended a face-to-face information session administered by a community health nurse and received a booklet that contained food calorie chart and strategies and recommendations for weight management, including understanding calories, portion control, replacement and substitution, volumetrics eating plan (eating foods that are healthy to make one feel full on fewer calories), strategies for healthy cooking, determining personal barriers for weight management and overcoming them, setting realistic goals and making commitment, self-monitoring, techniques for healthy eating at work, strategies for eating out, creating healthy food and eating environments, and overcoming emotional eating.

 Before intervention, each participant's cell phone was checked for sending and receiving Persian messages. All subjects in experimental group knew how to send and receive SMS through their own cell phone. The experimental group received text messages about weight management twice a day for two months. In addition, they were instructed to weekly self-weight by a digital calibrated scale which was left in their work place and to send the data to the principle researcher via SMS as well; the principle researcher compared each week's weight with the previous amount and sent them feedback. Therefore, the experimental group received a feedback of weekly self-monitored information. The control group did not receive any SMS and was not instructed to weekly self-weight during the study. After three months, all subjects were measured for secondary weight in a standardized manner by the same community health nurse, and the data were compared.

### 2.3. Messages Characteristics

 We used a web-based SMS service to send and receive messages. In this system, the principle researcher could check the status of each sent item. If a message failed to be delivered, it was sent again to the recipient. If the recipient still could not receive the message, the principle researcher called her to investigate the reason. Unlike SMS size in Persian alphabets and its limited characters, web-based service provides an opportunity of sending and receiving longer messages without breaking them. As many cell phones cannot send or receive MMS, we could not use multimedia messaging service. The intervention was designed in various topics and included strategies for weight loss such as understanding and controlling weight gain barriers, food substitutions, finding inner motivation, setting realistic goals, changing perspectives, enjoying healthier foods, healthy eating strategies, and making healthy meals. No specific instruction on diet was given to either group because the program was aimed to provide a supportive, self-directed weight management strategy. Messages were not based on a theoretical model, but they had an educational, supportive, and positive reinforcement or encouragement content. All participants received the same set of messages in the same order. To prevent repetition and to make the intervention attractive, the subjects never received the same message twice. About one-quarter of sent messages required a reply. Those messages were sent every two days. These messages mostly consisted of open-ended questions that the principle researcher provided them with customized feedbacks. Some examples of sent messages are as follows:

*Tip or Recommendation Messages (General Message)*

 Separate eating from your other emotions. Avoid eating when you are nervous, anxious, or depressed.
 Eat slowly and calmly and chew 20 times per spoonful.
 Avoid eating meals in front of the computer or TV and try to concentrate on what you are eating.
 Think about the total calories you consume.

*Question Messages (Interactive Message)*

 How many times you have skipped breakfast last week? Participant answered 3 times, feedback: You have missed breakfast last week. Eating early every day keeps you from overeating during the day.
 Remember to weight yourself this morning Participant answered, feedback: excellent work! You have lost [*x*] this week. Think of your plan for next week; you can do it!
 What did you eat for snacks today? Participant answered: I had a bag of potato chips, feedback: reduce your calorie intake by leaving out “unnecessary foods” high in sugar and/or fat; you can replace it by broccoli, carrot, or other yummy veggies.

### 2.4. Statistical Analysis

The data were analyzed using SPSS software (version 17, SPSS Inc., Chicago, IL). The homogeneity of demographic and baseline data between the experimental and control groups was examined through chi-square, *t*- and Fisher's exact tests. Mann-Whitney test was used to examine weight and BMI changes between baseline and 3 months in both groups. In all tests, differences were considered statistically significant if the *P* value was less than 0.05. 

## 3. Results

A total of 5200 SMSs were sent during two months (including both general and interactive messages); 3% of sent messages failed to deliver. The most important reason for failing was full Inbox. In that case, the subject was asked to empty her cell phone Inbox. 

Of the study population, 73 subjects completed the entire study, while 8.75% of women did not complete the entire study. In the experimental group, one subject became pregnant, and two subjects began taking medications known to cause weight changes. In the control group, two subjects entered in a special diet and exercise program, and two subjects refused to complete the secondary assessment. Statistical analysis showed no difference in sample characteristics between all subjects in the experimental and control groups (Table 1). Statistical analysis showed no significant difference between both groups in weight before the study. The subjects in the control group averaged 72.42 kg at baseline and lost a mean of 0.69 ± 1.23 in three months. The subjects in the experimental group averaged 74.32 kg at baseline; after 3 months, they lost a mean of 2.19 ± 03.06 kg. Statistical analysis revealed significantly more weight loss for the experimental than the control groups. The experimental group lost 1.5 kg more than the control group (*P* = 0.018). The weight loss was equal to 2.94% and 0.95% of their initial weight in experimental and control groups, respectively (Table 2). The mean reduced BMI was significantly greater in the intervention group: 0.83 ± 1.16 (kg/m) in the intervention group and 0.26 ± 0.48 (kg/m^2^) in the control group (*P* = 0.037). These equate to the 2.9% and 0.9 reduced BMI in the experimental and control group, respectively (Table 3). 

## 4. Response Rate

In average, each subject in the experimental group received 130 messages. One-quarter of messages need a reply. The average response rate to the messages that requested a reply was relatively low (38%) which dropped considerably over 2 months. During the first week, they responded to 50% of sent messages that requested a reply, but by the week 8, response rate decreased to 20%. 

## 5. Discussion

This pilot study suggests cell phone as a useful tool which helps individuals lose weight. Our results are consistent with a text message-based randomized controlled trial which reported 3.16% of initial body weight loss through 3 interventions: daily text messages, monthly paper materials, and brief phone calls [9]. As women have good motivation to control their body weight [19] and all of our participants and most of participant in other similar studies were women [9, 17, 20], further studies are needed on men to confirm our findings. Beside, in the present study, samples were employed healthy urban volunteer women; future studies are needed to investigate the effect of similar intervention on other female groups such as unemployed women, those with weight related comorbidities, rural women, and nonvolunteer participants. This study lasted for three months, two months intervention and one month wash-out period. The two-month period of study is short and inadequate to determine if a more clinically important achievement could be accomplished; longer-term studies are needed to investigate the effects of text messaging on weight management and weight maintenance.

 In several studies, the overall response rate to messages was low [9, 13]. In one study, the average response rate was 42/8% and steadily dropped over time [13]. Consequently, we designed the current study to have fewer messages (a quarter) which need a reply, but in the present study, still the average response rate of the interactive messages was considerably low (38%), and more studies should be done to investigate the effect of relevant factors on the acceptability and the interactivity of message-based interventions on health. Text-messaging may be superior to the Internet-based programs in Iran because the Internet penetration rate is low in Iran. Also, via text messaging, we can share the information to the subjects without logging into the Internet. In addition, addressing obesity with an effective, acceptable, and economic approach is necessary in developing countries where financial resources are limited. In this study, cell phone-based intervention using SMS effectively lowered the body weight and BMI of Iranian overweight women. Because of very low price, SMS seems to be a cost-benefit communication tool for health behavior interventions. 

For the future development and integration of short message service into weight management programs in a broad population, some considerations should be taken into account; it is of crucial importance to consider clients as individual with individual needs, and preferences. A high degree of customization is needed to provide clients with services with individualized approach in which the weight management plan is formulated according to each patient's characteristics, needs, and preferences. In addition, supplementary components such as e-mail and the Internet can be integrated with SMS because of some limitations of short message service such as limited size of SMS or small memory and screen size of mobile phone. A potential drawback of the use of short message service in weight management programs in a broad population is potential marginalization of certain populations, such as those who are illiterate or have not mobile phone; however, those limitations have been reduced as mobile technology advanced and became more widespread, for example, technologies exist that provide voice and pictures instead of text for those with limited literacy.

Our study had several limitations: the sample was volunteer, employed, urban, and specialized with narrow age range, in which our results cannot be generalized to other Iranian citizens. Further studies in large populations should be done to determine the optimal frequency and intensity of text messaging services, to engage participants, and to assess the effect of sociodemographic variables on response rate, adherence, and changes in weight. 

In conclusion, cell phone text messaging is a brief, available, economic, and communicative tool with potential usage in health related interventions especially in developing countries where other information resources are restricted. Interventions longer than two months on a broad population would necessitate repetition to determine if a more clinically important achievement could be accomplished. More studies are necessary to investigate what factors affect acceptability, interactivity, applicability, and utility of SMS for health related interventions in Iran. 

## Figures and Tables

**Table 1:** Baseline characteristics by groups, overweight and obese employed women.

Characteristics	Experimental group	Control group	Total	*P* value
Age (mean ± SD)	37.1 ± 7.4	38 ± 7.4	37.55 ± 7.66	0.34*
BMI (mean ± SD)	28.15 ± 3.1	28.37 ± 3.43	28.26 ± 3.2	0.76*
Weight (kg)	74.32 ± 9.37	72.42 ± 9.11	73.68 ± 9.7	0.37*
Educational status				
High school diploma	11 (27.5%)	8 (20%)	19 (23.8%)	0.56**
College degree/baccalaureate	27 (67.5%)	28 (70%)	55 (68.8%)	
Graduate degree	2 (5%)	4 (10%)	6 (7.5%)	
Marital status				0.87**
Single	10 (32.5%)	13 (16.25%)	(28.75%)	
Married	30 (76.5%)	27 (69.2%)	(71.25%)	

*t-test.

**Chi-square test.

**Table 2:** Effect of the intervention on body weight.

Weight group	Baseline weight (kg) (mean ± SD)	After three months weight (kg) (mean ± SD)	Weight loss (kg) (mean ± SD)	Weight loss (Percentage of initial weight) %	Mann-Whitney test for weight loss in three months
Experimental	74.32 ± 9.73	72.31 ± 9.87	2.19 ± 3.06	2.94	*P* = 0.018* *Z* = −2.37
Control	72.42 ± 9.11	72.13 ± 9.45	0.69 ± 1.23	0.95	

*The experimental group significantly lost more weight than the control group.

**Table 3:** Effect of the intervention on body mass index.

BMI	Baseline BMI (kg/m^2^) (mean ± SD)	After 3 months BMI (kg/m^2^) (mean ± SD)	Reduction in BMI (mean ± SD)	Reduction in BMI (Percentage of initial BMI) %	Mann-Whitney test for reduction in BMI at 3 months
Experimental	28.15 ± 3.1	27.39 ± 3.04	0.83 ± 1.16	2.9	*P* = 0.037* *Z* = 1.41
Control	28.37 ± 3.34	28.25 ± 3.57	0.26 ± 0.48	0.9	

*The mean reduction in BMI was 0.57 (kg/m^2^) more than in the experimental group (*P* = 0.037).

## References

[1] Kelly T, Yang W, Chen C-S, Reynolds K, He J (2008). Global burden of obesity in 2005 and projections to 2030. *International Journal of Obesity*.

[2] Bahrami H, Sadatsafavi M, Pourshams A (2006). Obesity and hypertension in an Iranian cohort study; Iranian women experience higher rates of obesity and hypertension than American women. *BMC Public Health*.

[3] Haslam DW, James WPT (2005). Obesity. *The Lancet*.

[4] Simon GE, Ludman EJ, Linde JA (2008). Association between obesity and depression in middle-aged women. *General Hospital Psychiatry*.

[5] Wadden TA, Butryn ML, Sarwer DB (2006). Comparison of psychosocial status in treatment-seeking women with class III versus class I-II obesity. *Obesity*.

[6] WHO Global database on body mass index. http://apps.who.int/bmi/index.jsp?introPage=intro_3.

[7] WHO Noncommunicable Disease Surveillance WHO Global NCD Infobase. http://www.who.int/ncd_surveillance/.

[8] Kelishadi R, Alikhani S, Delavari A, Alaedini F, Safaie A, Hojatzadeh E (2008). Obesity and associated lifestyle behaviours in Iran: findings from the first national non-communicable disease risk factor surveillance survey. *Public Health Nutrition*.

[9] Patrick K, Raab F, Adams MA (2009). A text message-based intervention for weight loss: randomized controlled trial. *Journal of Medical Internet Research*.

[10] Fjeldsoe BS, Marshall AL, Miller YD (2009). Behavior change interventions delivered by mobile telephone short-message service. *American Journal of Preventive Medicine*.

[11] Cole-Lewis H, Kershaw T (2010). Text messaging as a tool for behavior change in disease prevention and management. *Epidemiologic Reviews*.

[12] Zolfaghari M, Mousavifar SA, Pedram S, Haghani H (2012). The impact of nurse short message services and telephone follow-ups on diabetic adherence: which one is more effective?. *Journal of Clinical Nursing*.

[13] Harris LT, Lehavot K, Huh D (2010). Two-way text messaging for health behavior change among human immunodeficiency virus-positive individuals. *Telemedicine Journal and E-Health*.

[14] Koshy E, Car J, Majeed A (2008). Effectiveness of mobile-phone short message service (SMS) reminders for ophthalmology outpatient appointments: observational study. *BMC Ophthalmology*.

[15] Vilella A, Bayas J-M, Diaz M-T (2004). The role of mobile phones in improving vaccination rates in travelers. *Preventive Medicine*.

[16] Free C, Knight R, Robertson S (2011). Smoking cessation support delivered via mobile phone text messaging (txt2stop): a single-blind, randomised trial. *The Lancet*.

[17] Hurling R, Catt M, de Boni M (2007). Using internet and mobile phone technology to deliver an automated physical activity program: randomized controlled trial. *Journal of Medical Internet Research*.

[18] Soureti A, Murray P, Cobain M, Chinapaw M, van Mechelen W, Hurling R (2011). Exploratory study of web-based planning and mobile text reminders in an overweight population. *Journal of Medical Internet Research*.

[19] Joo N-S, Kim B-T (2007). Mobile phone short message service messaging for behaviour modification in a community-based weight control programme in Korea. *Journal of Telemedicine and Telecare*.

[20] Haapala I, Barengo NC, Biggs S, Surakka L, Manninen P (2009). Weight loss by mobile phone: a 1-year effectiveness study. *Public Health Nutrition*.

